# An Ocular Emergency Often Ignored

**DOI:** 10.7759/cureus.41754

**Published:** 2023-07-12

**Authors:** Shashank Cheemalapati, Suresh M Babu

**Affiliations:** 1 Internal Medicine, JSS Medical College and Hospital/JSS Academy of Higher Education & Research (JSSAHER), Mysore, IND

**Keywords:** radial artery occlusion (rao), retinal artery occlusion, emergency, ocular, branched retinal artery occlusion

## Abstract

Branch retinal artery occlusion (BRAO) is a relatively rare vascular disorder characterized by the occlusion of one or more branches of the central retinal artery, with an incidence of around 5 per 100,000 persons per year. Its ability to cause permanent vision loss in a specific visual field makes it a significant clinical obstacle that requires careful management. We describe a case of a 77-year-old male patient, a known hypertensive (on medication and whose hypertension was under control), presented with sudden painless loss of vision in the superior field of his right eye. Timely and accurate diagnosis is crucial to initiate appropriate management. Patients with hypertension should be routinely screened for its various micro- and macrovascular complications, and prophylactic therapy/management should be started wherever warranted.

## Introduction

Branch retinal artery occlusion (BRAO) is a retinal vascular disorder that can lead to irreversible visual impairment. It occurs due to the occlusion of one or more branches of the central retinal artery, resulting in a compromised blood supply to the affected retinal segments [1]. Although less prevalent than central retinal artery occlusion (CRAO), BRAO poses significant therapeutic challenges to clinicians due to its ability to cause permanent visual loss in the affected visual field.

BRAO etiology is multifactorial, with atherosclerosis, emboli, vasculitis, and hypercoagulable states among the leading causes. Atherosclerosis, often involving the carotid arteries, is a common underlying condition contributing to BRAO. Emboli originating from sources such as the carotid arteries and other sources, such as platelet-fibrin emboli and calcific cardiogenic emboli, can obstruct retinal artery branches and result in BRAO. Vasculitis, including conditions such as giant cell arteritis and Takayasu arteritis, can lead to occlusion of retinal arteries. Hypercoagulable states, such as antiphospholipid syndrome and thrombophilia, may also increase the risk of BRAO [1,2].

In this case report, we discuss a case of a 77-year-old male patient, a known hypertensive, who presented with sudden painless visual loss with a visual field defect in the right superior field.

## Case presentation

A 77-year-old male, who is a known Stage II hypertensive for 10 years (on treatment with an angiotensin-receptor blocker, and compliant with medication), presented to the emergency department with a history of sudden onset, painless loss of vision in his right eye. The patient was asymptomatic three days before admission when he developed a sudden loss of vision affecting his superior field of vision in his right eye while he was performing his routine daily activities. The patient also complained of numbness and tingling in his right arm. He did not have any complaints of limb weakness, headache, loss of consciousness, giddiness, seizures, or any involuntary bowel/bladder movements. The patient was a known case of benign prostatic hyperplasia and was on treatment with a tamsulosin + finasteride combination for the same.

On examination, the patient was afebrile and tachycardic with a pulse rate of 100 bpm with no radio-radial and/or radio-femoral delay. The patient presented with a blood pressure of 180/90 mmHg. Cardiovascular, respiratory, and abdominal examinations were normal. On cranial nerve examination, the patient was found to have a loss of vision in the upper half of the field of vision of his right eye, whereas the left eye had a normal full field of vision. Relative afferent pupil defect was found to be positive for the right eye. The visual acuity of the right eye in the lower field was normal (6/6) but was nil in the upper field. The patient’s left eye had a normal full visual acuity of 6/6. The remaining neurological examination was normal, and the patient had no other focal neurological defects.

**Figure 1:**
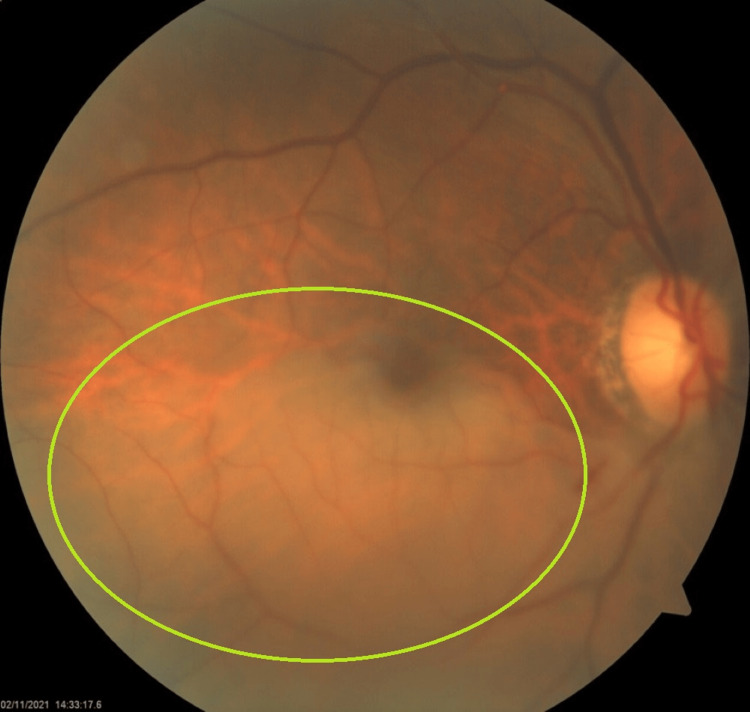
Fundus examination revealed retinal whitening seen in the infratemporal retina involving the inferior part of the foveal avascular zone, with the disc appearing normal in color, size, shape, and margin

Fundus examination revealed retinal whitening seen in the infratemporal retina involving the inferior part of the foveal avascular zone, with the disc appearing normal in color, size, shape, and margin, as seen in Figure 1.

No other signs of end-organ damage (papilledema, flame hemorrhages, acute kidney injury) were observed. The patient was subsequently started on a calcium-channel blocker (CCB) & angiotensin-receptor blocker (ARB) combination anti-hypertensive. The opinion of an ophthalmologist was sought, and the patient was prescribed tab. Diamox 250 mg TID with Dorzox T drops BD.

The patient underwent carotid Doppler and was found to have a diffuse atherosclerotic disease of bilateral carotid arteries with an eccentric calcified plaque measuring 8 x 2.1 mm (length x thickness) in the left carotid bulb, causing 10-15% luminal narrowing with no significant hemodynamic changes.

**Figure 2:**
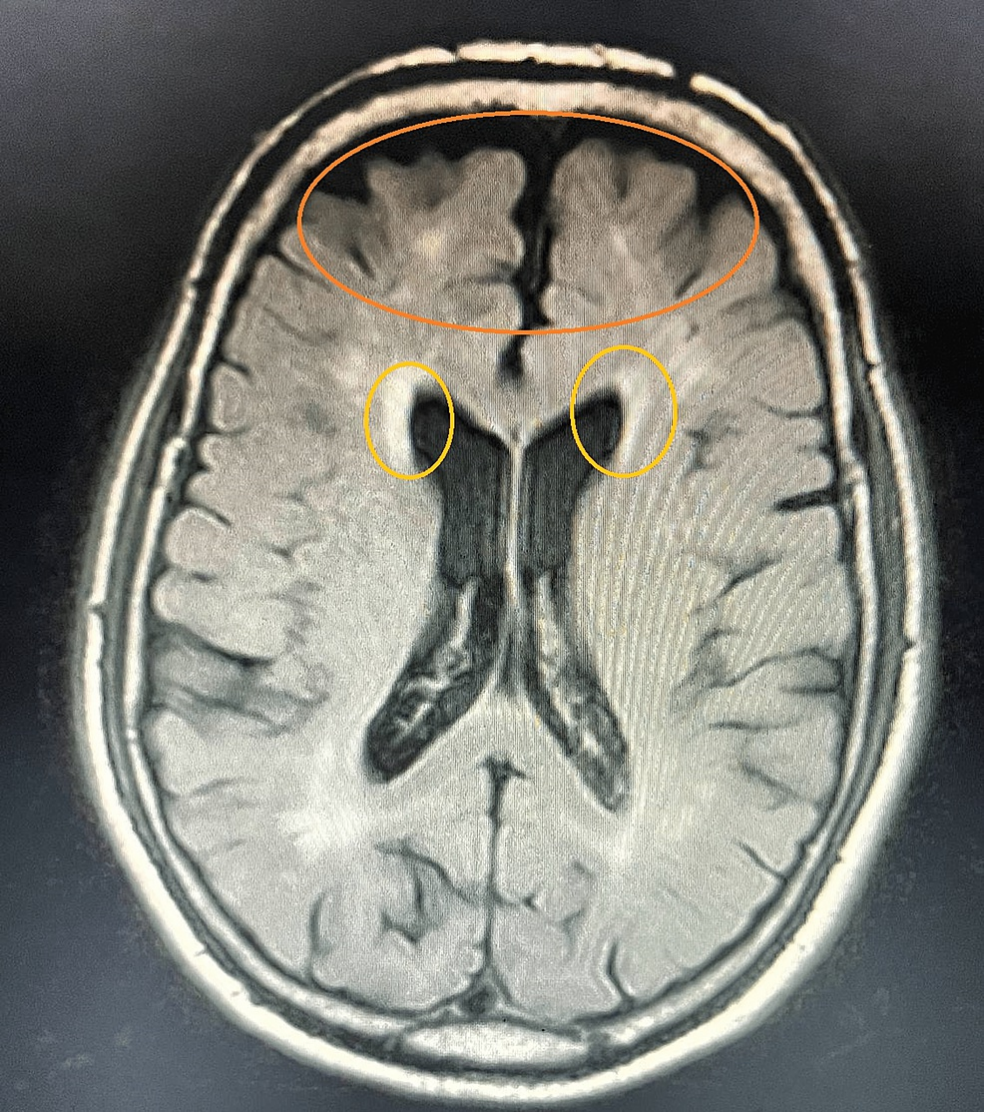
The orange circle shows changes representative of age-related cerebral atrophy, and the yellow circle show hyperintense areas with chronic small vessel ischemic changes

Lipid profile showed elevated levels of direct LDL cholesterol at 149 mg/dl (reference value ~ 99 mg/dl) and mildly elevated triglyceride levels of 154 mg/dl (reference value ~ 150 mg/dl). The patient was then started on statins - tab. Rozucor ASP 10 mg BD in view of the same, along with dual anti-platelet therapy (aspirin 75 mg + clopidogrel 75 mg HS). Hypercoagulable state workup was done, and the corresponding levels were within normal limits, hence ruling out hypercoagulability as the cause. ESR levels were also within normal limits at 10 mm/hr (normal range of 0-15 mm/hr in men). Echocardiography showed aortic valve sclerosis, with mild concentric LVH. A neurologist's opinion was taken, and MRI Brain was done, which showed age-related cerebral atrophy with chronic small vessel ischemic changes, as seen in Figure 2 below.

The patient was diagnosed to have BRAO involving the right inferior branch of the right retinal artery. The patient’s blood pressure was under control at the time of discharge, recorded at 126/80 mmHg. The patient was advised to continue the newly prescribed CCB-ARB combination (tab. Triolmesar 20 mg OD), T=tab. Deplatt CV 10 mg HS (dual antiplatelet + statin combination), tab. Diamox 250 mg TID, and Dorzox T drops BD. The patient was followed up at seven days post discharge, followed by regular follow-up appointments at three, six, and 12 months. At six months post discharge, the patient was found to have regained a visual acuity of 20/40 in the superior field of his right eye.

## Discussion

Retinal artery occlusion is an ophthalmic emergency similar to an ischemic stroke. It presents with sudden onset monocular vision loss [3]. Retinal artery occlusions are commonly caused by emboli of atheromatous origin from the ipsilateral carotid artery [4]. Other common causes of retinal artery occlusion include cardiogenic embolism, hypercoagulable states, and carotid artery dissection [5]. The occlusion can be either in the central retinal artery or any of its branches.

**Figure 3:**
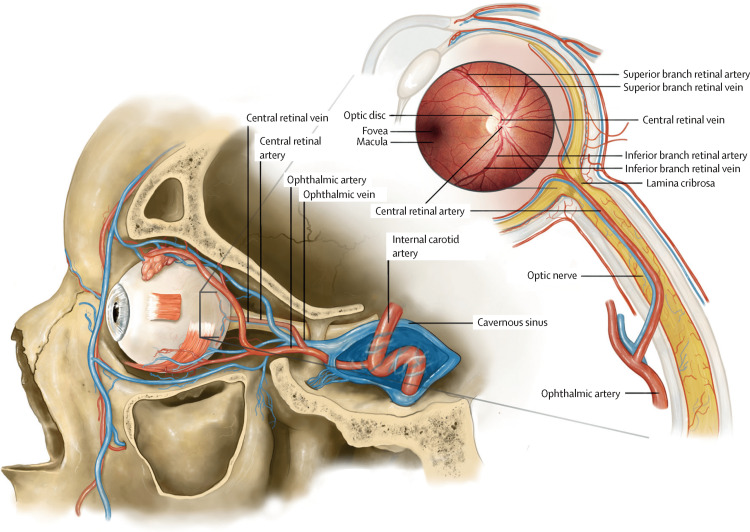
Diagrammatic representation showing the arterial supply and venous drainage of the retina Reproduced from Scott, Ingrid U [7] DOI : 10.1016/S0140-6736(20)31559-2

BRAO is a distinct form of retinal vascular occlusion characterized by the blockage of one or more branches of the central retinal artery. It presents a unique clinical profile compared to CRAO due to the involvement of specific retinal segments - thereby leading to the loss of vision, which is restricted to a section of the visual field [1]. The central retinal artery, which is a branch of the ophthalmic artery, is the main vessel supplying the retina. The central retinal artery divides into superior and inferior branches, which in turn divide into temporal and nasal branches, thus supplying all four quadrants of the retina [6], as seen in Figure 3 below. The above fundus picture (Figure 1) reflects the occlusion of the inferotemporal branch of the central retinal artery.

A prompt workup is essential to determine the underlying etiology. Diagnostic tools, such as optical coherence tomography (which can visualize the inner retinal atrophy and loss of inner retinal architecture), fluorescein angiography (wherein the retrograde filling of retinal arterial/venous branches is indicative of spontaneous healing) [8], and carotid imaging aid in confirming the diagnosis and identifying potential sources of emboli or vascular abnormalities [9].

Management of retinal artery occlusion involves measures to immediately restore circulation, prevent neo-vascular complications to the eye, and prevent any further ischemic events to the eye or any other end organs. Treatment strategies include addressing underlying risk factors, such as atherosclerosis, vasculitis, or hypercoagulable states. Anticoagulation therapy may be considered in selected cases, and intravenous thrombolysis can be utilized in acute presentations. However, they have their own risks, with an increased risk of intraocular hemorrhage and other systemic hemorrhagic complications - such as intracranial hemorrhage (especially in patients with comorbidities or a high bleeding risk). Novel interventions, such as selective retinal artery fibrinolysis and retinal laser photocoagulation, show promise in limited studies [10,11]. However, the visual prognosis in BRAO remains guarded, with around 90% of patients seeing better than 20/40 over the course of a few weeks [1]. 

The other differential diagnoses that could be considered in a patient with sudden onset monocular vision loss include retinal detachment, anterior ischemic optic neuropathy, and branch retinal vein occlusion. Anterior ischemic optic neuropathy presents with sudden onset of monocular vision loss. Fundus examination shows initially an oedematous disc that pales later [12]. Retinal vein occlusions are also ocular emergencies with sudden onset of visual disturbances, with fundus pictures showing dilated and tortuous veins with areas of edema and hemorrhage around them [13]. Retinal detachment can present with sudden onset of vision disturbances with funduscopic images revealing greying of the detached area of the retina along with tears in most patients [14].

## Conclusions

BRAO is a relatively rare vascular disorder that requires prompt diagnosis and management to optimize visual outcomes. The use of diagnostic modalities, such as fundoscopy and carotid artery Doppler, aided in accurately localizing the occlusion. Treatment strategies aimed at addressing underlying risk factors, including anticoagulation therapy, resulted in the stabilization of the patient's vision and the prevention of further occlusive events. However, the prognosis for visual recovery in BRAO remains guarded, with the need for ophthalmological follow-up at regular intervals. Further studies are warranted to explore novel interventions and improve therapeutic approaches to enhance visual outcomes in this challenging ocular condition.
